# *Heterodera schachtii* Tyrosinase-like protein - a novel nematode effector modulating plant hormone homeostasis

**DOI:** 10.1038/s41598-017-07269-7

**Published:** 2017-07-31

**Authors:** Samer S. Habash, Zoran S. Radakovic, Radomira Vankova, Shahid Siddique, Petre Dobrev, Cynthia Gleason, Florian M. W. Grundler, Abdelnaser Elashry

**Affiliations:** 1 Rheinische Friedrich-Wilhelms-University of Bonn, INRES – Molecular Phytomedicine, Karlrobert-Kreiten-Straße 13, D-53115 Bonn, Germany; 2 Institute of Experimental Botany AS CR, Rozvojová 263, CZ-16502 Prague 6, Czech Republic; 3 Washington State University, Dept. of Plant Pathology, Pullman WA, 99164-6430 335 Johnson Hall USA

## Abstract

The beet cyst nematode *Heterodera schachtii* causes major yield losses in sugar beet. Understanding the interaction between *H. schachtii* and its host plant is important for developing a sustainable management system. Nematode effectors play a crucial role in initializing and sustaining successful parasitism. In our study, we identified a gene (*Hs-Tyr*) encoding a tyrosinase functional domain (PF00264). We describe *Hs-Tyr* as a novel nematode effector. *Hs-Tyr* is localized in the nematode esophageal gland. Up-regulation of its expression coincided with the parasitic developmental stages of the nematode. Silencing *Hs-Tyr* by RNA interference made the treated nematodes less virulent. When RNAi-treated nematodes succeeded in infecting the plant, developing females and their associated syncytial nurse cells were significantly smaller than in control plants. Ectopically expressing the *Hs-Tyr* effector in Arabidopsis increased plant susceptibility to *H. schachtii*, but not to the root-knot nematode *Meloidogyne incognita*. Interestingly, *Hs-Tyr* in the plant promoted plant growth and changed the root architecture. Additionally, the expression of *Hs-Tyr* in Arabidopsis caused changes in the homeostasis of several plant hormones especially auxin and the ethylene precursor aminocyclopropane-carboxylic acid.

## Introduction

Plant parasitic nematodes cause massive yield losses in many important crops and are therefore considered as a major problem in crop production^1^. The beet cyst nematode *Heterodera schachtii* is an important sedentary parasite of sugar beet^2^. When conditions are favorable, the infective second stage juveniles (J2s) hatch from the eggs and spread in the soil. Having reached a host root, they enter it and migrate through the cells into the vascular cylinder. There, they search for a specific cell that is suitable to establish the initial syncytial cell (ISC)^3, 4^. From ISC a highly active syncytial nurse cells system develops through the fusion of neighboring cells^5^. The hypertrophic and hypermetabolic syncytium serves as the only source of nutrients for the developing juveniles. The nematodes undergo three molts before reaching the adult stage; males leave the root in a vermiform shape, whereas females remain at the root, grow to a lemon-like shape, and finally rupture the root cortex. After mating, females produce eggs inside their bodies until they eventually die. After death, their cuticles turn into brown-tanned cysts containing the eggs.

Since the nematodes become sedentary upon feeding, they rely on their syncytia as the sole source of nutrition throughout their life cycle. Therefore, initiating and maintaining the syncytium is the key factor for successful parasitism. As a result, nematodes are equipped with effector proteins which support their parasitism by manipulating the host plant through suppression of plant defenses and the alteration of its developmental and physiological processes^3, 6, 7^.

Recent molecular studies have focused on the identification and characterization of effector proteins in order to understand their function in the plant-nematode interaction. Some effectors are able to modulate the plant defenses during the parasitism process, by either mimicking plant proteins or manipulating plant defenses. For example, a recent study found that the *H. schachtii* effector 4F01 is mimicking plant annexin and, by doing so, alters the host defenses against nematodes^8^. In another example, effector 10A06 has been shown to interact with spermidine synthase, and this interaction disturbs the host´s ability to produce defense-associated compounds such as salicylic acid^9^. According to a recent report, effector 30C02 binds and inhibits the pathogenesis related protein β-1,3-endoglucanase in the infected Arabidopsis plants, and thus, increases host susceptibility to nematode infection^10^. Nematode effectors can also affect basal immune responses. Basal defenses may be triggered by cell wall fragments produced during the nematode’s migration through the root or by unidentified PAMPs. However, *H. schachtii* venom-allergen like protein (VAP) acts as an apoplastic immune repressor which dampens the plant’s immune responses. Overexpressing VAP in Arabidopsis also increased plant susceptibility to unrelated pathogens, suggesting that it interferes with defense responses to different biotic stresses^11^.

In addition to effectors that seem to target plant defenses, nematodes are also able to produce molecules with hormone activity. These effectors enhance the plant’s physiological activities to the benefit of the nematodes. Recently, *H. schachtii* juveniles were shown to secrete cytokinins into the feeding site, which stimulate the cell division and growth needed for feeding site formation^12^. Additionally, nematodes secrete peptides that mimic the family of plant peptide hormones called CLEs, and this enables the nematodes to developmentally reprogram the root cells in order to initiate and maintain its feeding site^13^.

Based on an analysis of the *H. schachtii* transcriptome, we identified a novel candidate effector that encodes a nematode protein with a Tyrosinase domain (*Hs-Tyr)*. Tyrosinases are copper monooxygenases that catalyze the hydroxylation of monophenols and the oxidation of o-diphenols to o-quinols. As polyphenol oxidases that are involved in the formation of pigments such as melanin and other polyphenolic compounds. They exist in prokaryotes as well as in eukaryotes.

Within the *Hs-Tyr* sequence, we found a signal peptide but no transmembrane domain suggesting that it is a secreted protein. Here, we show that *Hs-Tyr* is transcribed in the esophageal gland and is required for successful nematode parasitism. Moreover, expressing the *Hs-Tyr* in Arabidopsis enhances the plants’ susceptibility to *H. schachtti*, promotes vegetative growth and induces hormonal changes, suggesting that *Hs-Tyr* affects plant growth and development to support nematode parasitism.

## Results

### Sequence domains prediction and phylogenetic analysis

**Figure 1:**
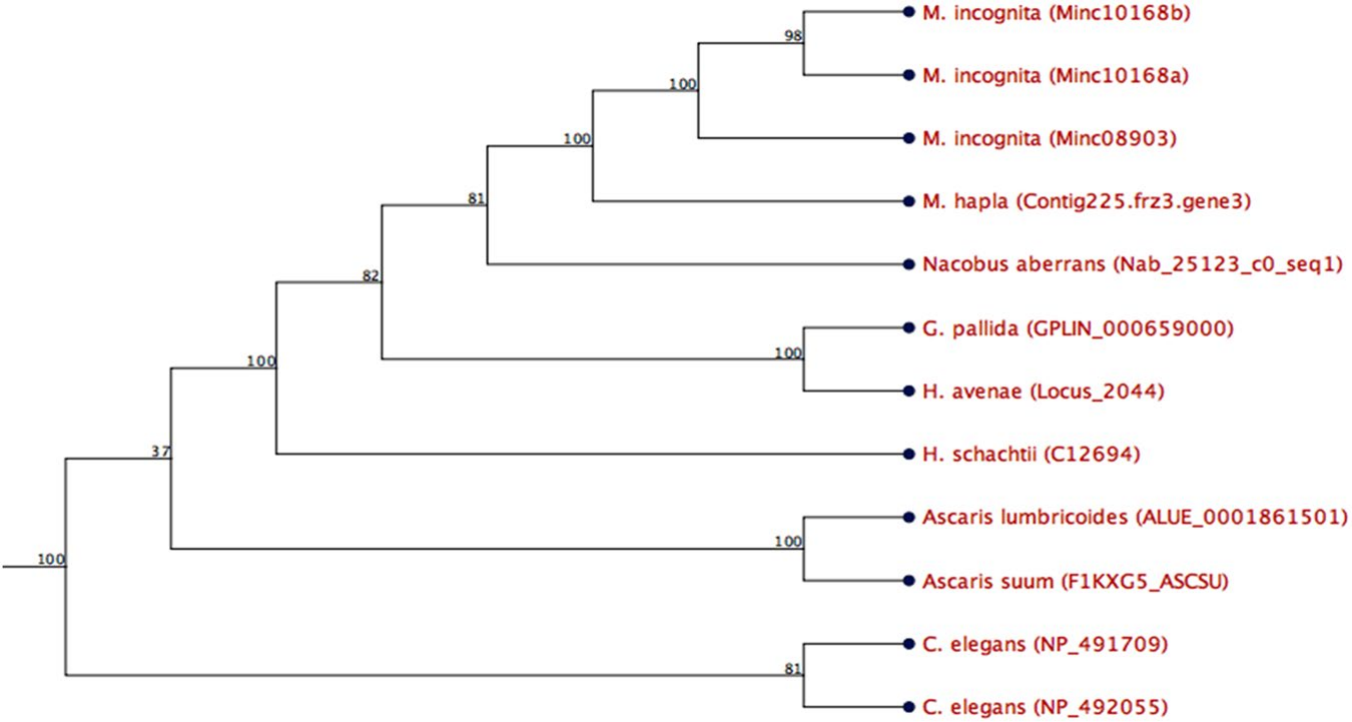
*Hs-Tyr* phylogenetic tree for tyrosinase-like genes of several nematode species. Tyrosinase-like gene from cyst nematodes cluster separately from root-knot nematodes among plant parasites, which, on the other hand, are separated from the animal-parasitic and free-living nematodes. Numbers on branches are the percentage of bootstrap (100 bootstrap).

The transcriptome analysis of *H. schachtii* J2s using next generation sequencing (Illumina) revealed a sequence that we designated *Hs-Tyr* (accession No. KU975565). It contains a tyrosinase domain (E-value: 3.2E-11) and four SHK domain like sequences (E-value: 2.2E-34) as predicted by Pfam domain analysis. Further sequence analysis predicted that the protein contains a signal peptide of 22 amino acids and lacks any transmembrane domains (Supplementary Fig. S1). Aligning *Hs-Tyr* to other tyrosinase-Like genes from several nematode species revealed a high level of sequence and structure similarity. In comparison to *Hs-Tyr*, Ce-tyr4 (*Caenorhabditis elegans*), Nab_25123_c0_seq. 1 (*Nacobus aberrans*), and GPLIN_000202700 (*Globodera pallida*) have the most similar organization of functional domains. The phylogenetic analysis of the nematode tyrosinase-like genes based on domain similarity showed that tyrosinases of plant parasites cluster separately from that of free-living and animal-parasitic nematodes. Furthermore, within the cluster of plant parasitic nematodes, the tyrosinases of the cyst- and root knot-nematodes cluster separately (Fig. 1).

### The *Hs-Tyr* localization and expression profile analysis

**Figure 2:**
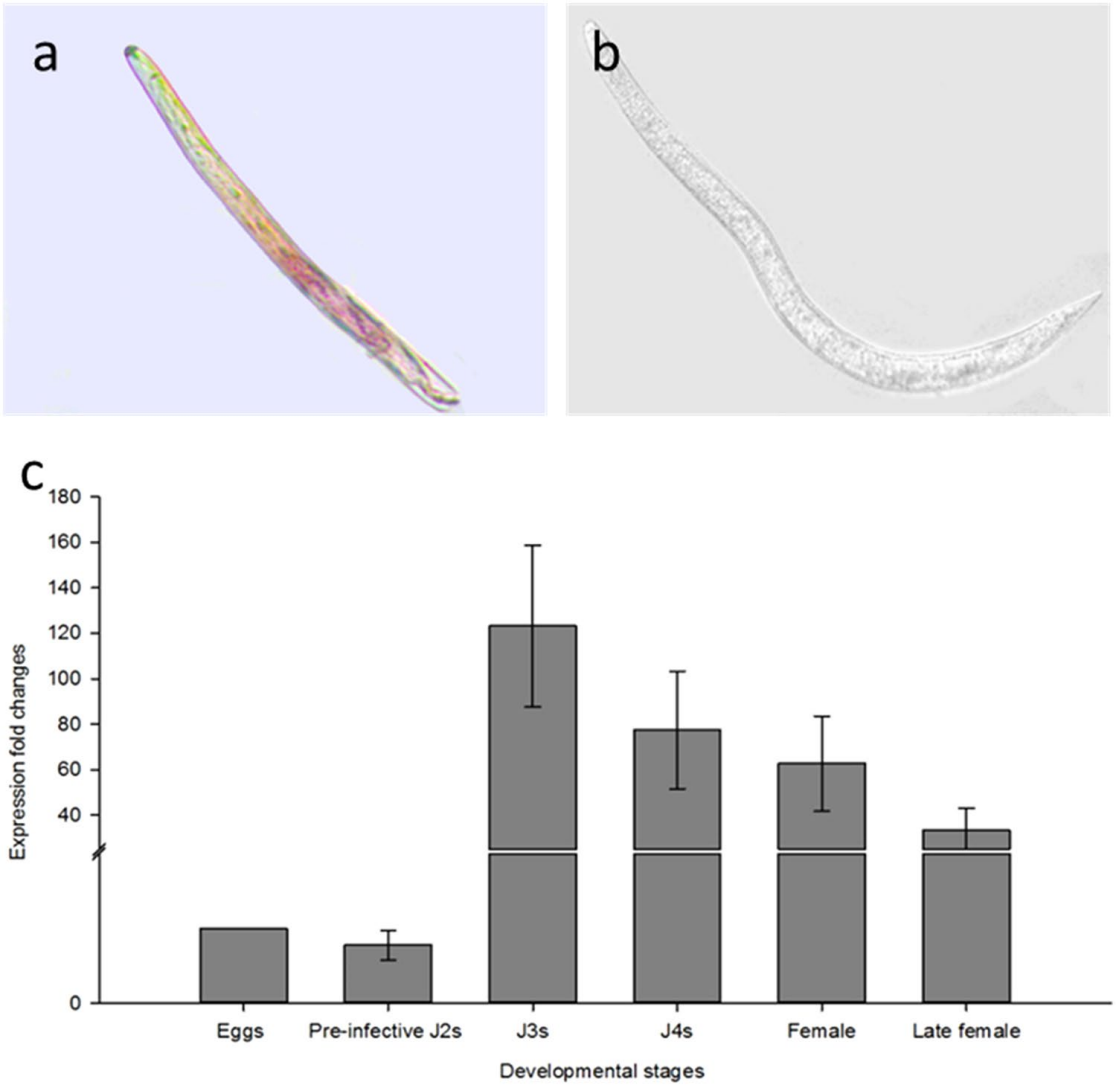
*Hs-Tyr* expression localization and profiling. (**a**) Localization of *Hs-Tyr* transcripts in the esophageal gland cells of *H. schachtii* J2s by whole mount *in situ* hybridization of digoxigenin-labeled antisense cDNA. (**b**) *In situ* hybridization negative control treated with digoxigenin-labeled sense probes showing no signals in the esophageal gland (Bar = 100 μm). (**c**) Relative mRNA expression levels of *Hs-Tyr* quantified by qPCR in six different life stages of *H. schachtii*. The fold change values were calculated and represent changes in mRNA level in pre-infective J2s, J3, J4, females and late females relative to that of eggs. Data are averages of three biologically independent experiments, each consisting of three technical replicates. *H. schachtii* actin was used as an internal control to normalize gene expression level.

To localize *Hs-Tyr* expression in the nematode body we performed a whole mount *in situ* hybridization on pre-infective J2s. In fact, the hybridized riboprobe was visualized within the esophageal gland cells of the J2s (Fig. 2a), while no signal appeared in the negative control using the sense probe (Fig. 2b). We further studied the expression profile of *Hs-Tyr* in correlation with the eggs, J2s, J3s, J4s, females and late females by qRT-PCR amplification using stage-specific cDNA. *Hs-Tyr* expression was found to be the lowest in eggs compared with the infective stages. The expression of *Hs-Tyr* was not significantly changed in pre-parasitic J2s, but there was a massive increase in expression in the later life stages when the nematodes had started feeding. The highest expression was in J3s. Later, gene expression decreased in J4s and female stages. The high expression of *Hs-Tyr* in the parasitic stages compared to the pre-parasitic stages suggests that it plays a role in parasitism (Fig. 2c).

### The effect of *Hs-Tyr* silencing and ectopic expression in the plant on nematode infection

**Figure 3:**
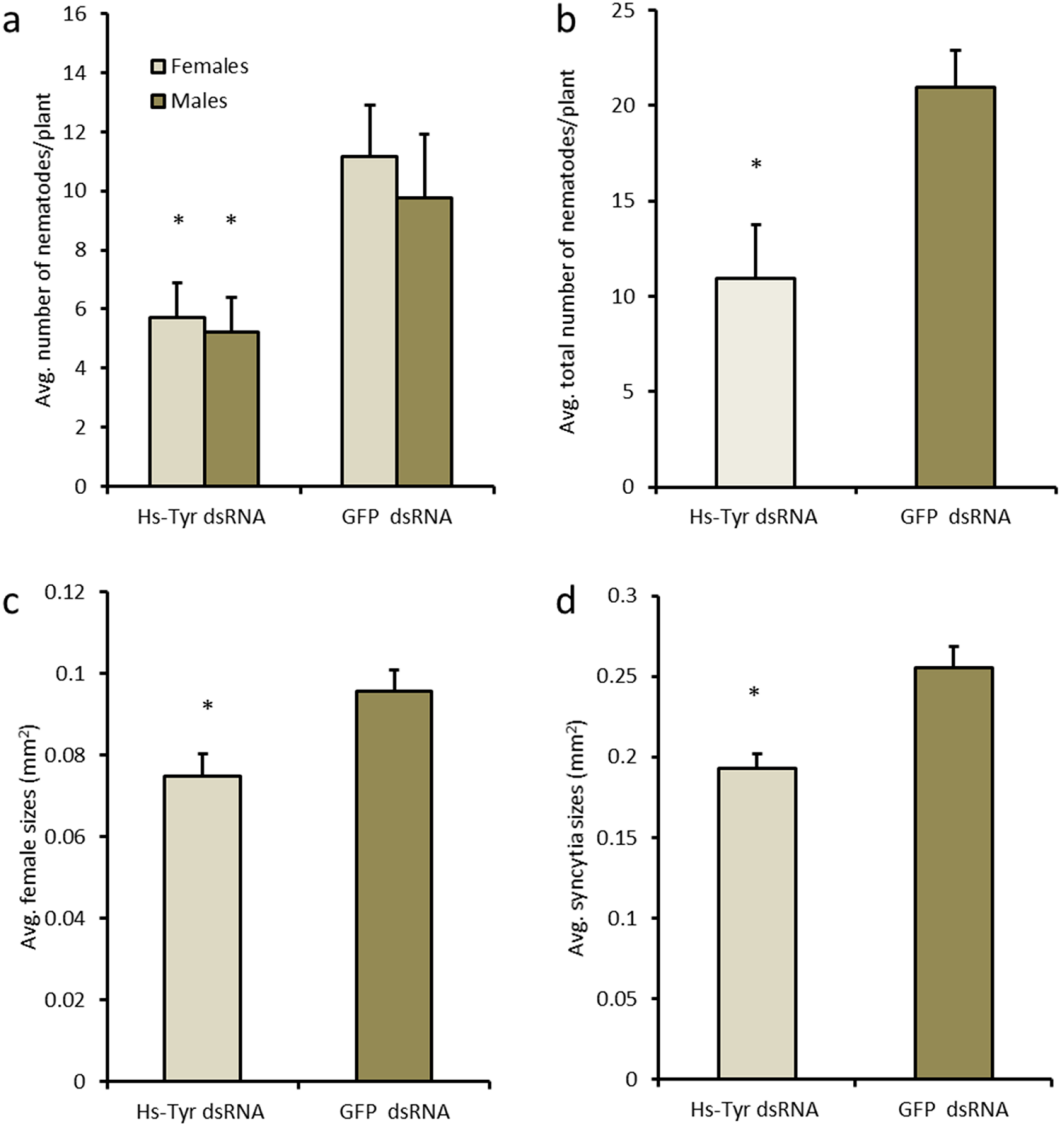
Effects of silencing *Hs-Tyr* on *H. schachtii* parasitism. The graphs show the results of bio-assays with J2 soaked in *Hs-Tyr* -specific dsRNA compared with J2 soaked in GFP dsRNA as a negative control. The following parameters were analysed: (**a**) number of males and females (**b**) total number of nematode infections (**c**) female size (**d**) size of syncytia. Data are based on three independent experiments. Each bar represents the mean ± standard error of n > 35. Asterisk indicate significant differences based on Student’s *t-test* (*P* < 0.05).

In order to analyze the role of *Hs-Tyr* for *H. schachtii* parasitism, we silenced *Hs-Tyr* in the nematodes by RNA-interference. Soaking the pre-parasitic J2s in *Hs-Tyr* dsRNA knocked down 80% of the endogenous *Hs-Tyr* transcript compared with nematodes treated with GFP dsRNA (Supplementary Fig. S2). In the next experiment, plants were inoculated with the dsRNA-treated nematodes. Soaked nematodes were clearly affected in their development. The numbers of males and females per plant (12 days after inoculation (DAI)) decreased significantly compared with nematodes soaked in the GFP dsRNA control (Fig. 3a). Total nematode infection decreased by 50% (Fig. 3b). The average female size was also reduced significantly compared with the GFP-treated nematodes (Fig. 3c). Syncytia associated with *Hs-Tyr* treated nematodes were significantly smaller, reaching a size of 0.2 mm^2^ compared with 0.27 mm^2^ in the control (Fig. 3d).

**Figure 4:**
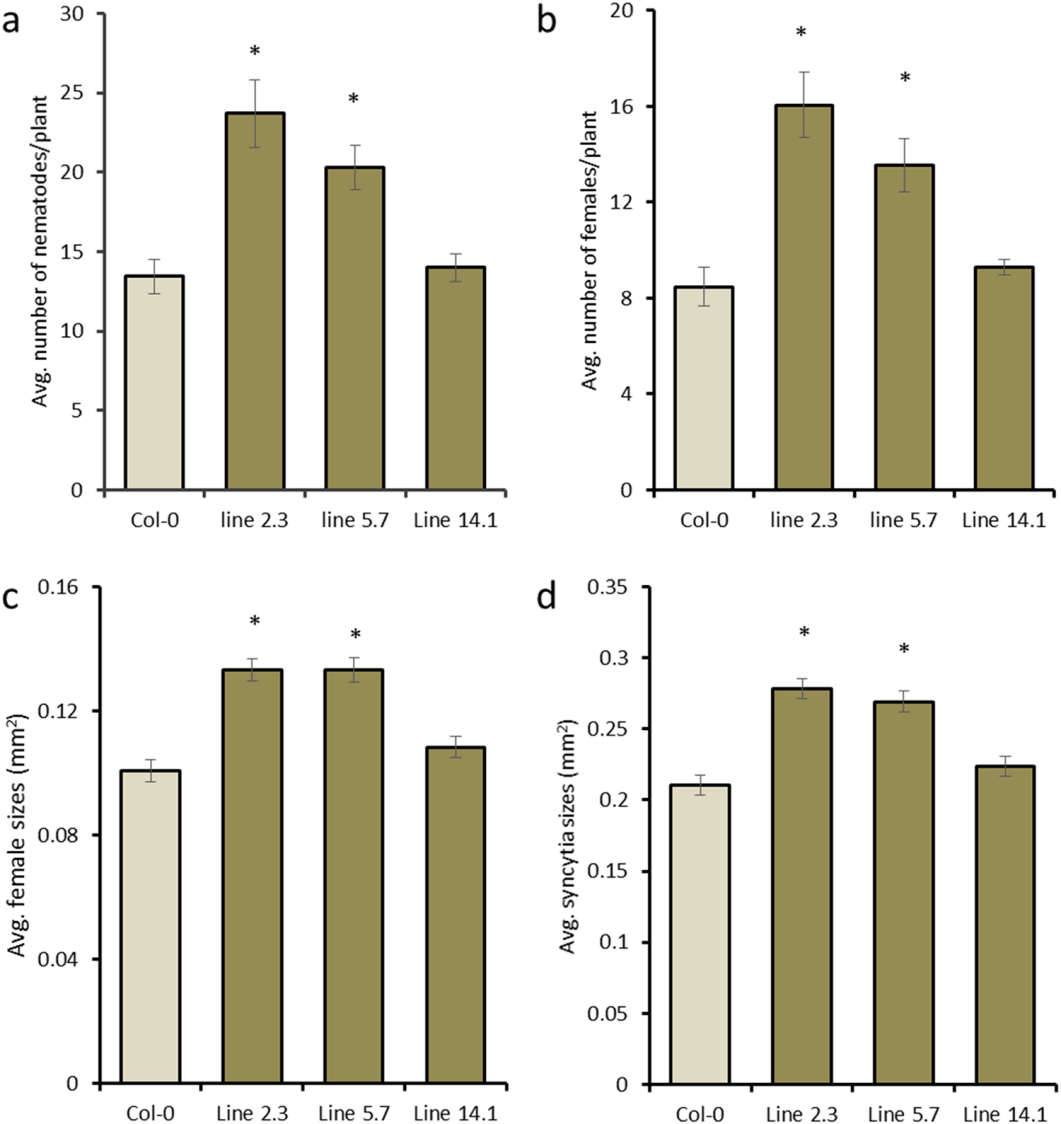
Effect of ectopic expression of *Hs-Tyr* on the development of *H. schachtii* in Arabidopsis. The following parameters were analysed: (**a**) total number of nematode infection per plant (**b**) number of females per plant (**c**) female size (**d**) syncytium size, compared with the wild type Col-0. Data are based on three independent experiments. Each bar represents the mean ± standard error of n > 35. Asterisks indicate significant differences based on Student’s *t-test* (*P* < 0.05).

In order to examine whether expressing *Hs-Tyr* in Arabidopsis affects susceptibility to *H. schachtii* infection, three separate transgenic Arabidopsis lines ectopically expressing 35 s::*Hs-Tyr* were infected with J2s. Recombinant expression of *Hs-Tyr* in the transgenic plants was confirmed and quantified using qPCR (Supplementary Fig. S3). The transgenic lines showed increased susceptibility to nematode infection. The total number of nematodes and the number of mature females increased significantly in lines 2.3 and 5.7 while no significant increase was found in line 14.1 (Fig. 4a and b). Similarly, the size of the mature females and their associated syncytia was increased significantly in the overexpression lines 2.3 and 5.7, while line 14.1 showed no significant difference (Fig. 4c and d). We then analyzed susceptibility of the transgenic plant lines to *M. incognita* and found that the number and size of galls did not differ from those of the wild type Col-0 (Supplementary Fig. S4).

### Ectopic expression of *Hs-Tyr* in Arabidopsis stimulates plant growth and modulates root architecture

**Figure 5:**
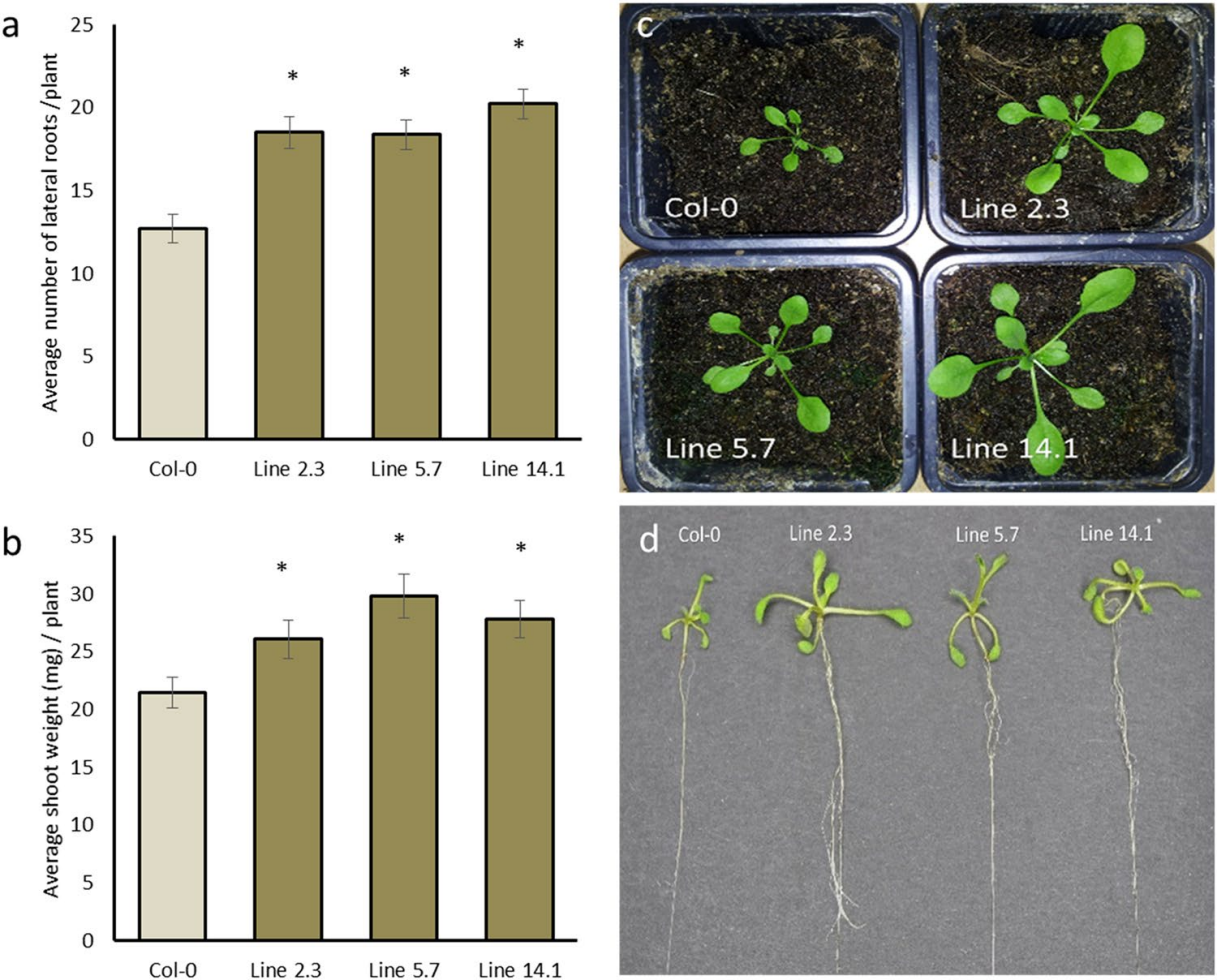
Effect of ectopic expression of *Hs-Tyr* on Arabidopsis growth. The following parameters were analysed: (**a**) number of lateral roots per plant (**b**) shoot weight and visible increase in the shoot growth. (**c**) and (**d**) comparison of the wild type Col-0 with the overexpression lines. Data are based on three independent experiments. Each bar represents the mean ± standard error of n = 27. Asterisks indicate significant differences based on Student’s *t-test* (*P* < 0.05).

Growth and development of the transgenic Arabidopsis plants ectopically expressing the *Hs-Tyr* were analyzed and compared with Col-0. The *Hs-Tyr* expressing lines did not show changes in root length and root weight compared with Col-0 (Supplementary Fig. S5), but the root architecture was significantly changed. The number of lateral roots compared with the Col-0 plants was higher (Fig. 5a) and the shoot weight and growth were significantly increased (Fig. 5b,c and d).

### Ectopic expression of *Hs-Tyr* triggers changes in plant hormone homeostasis

**Figure 6:**
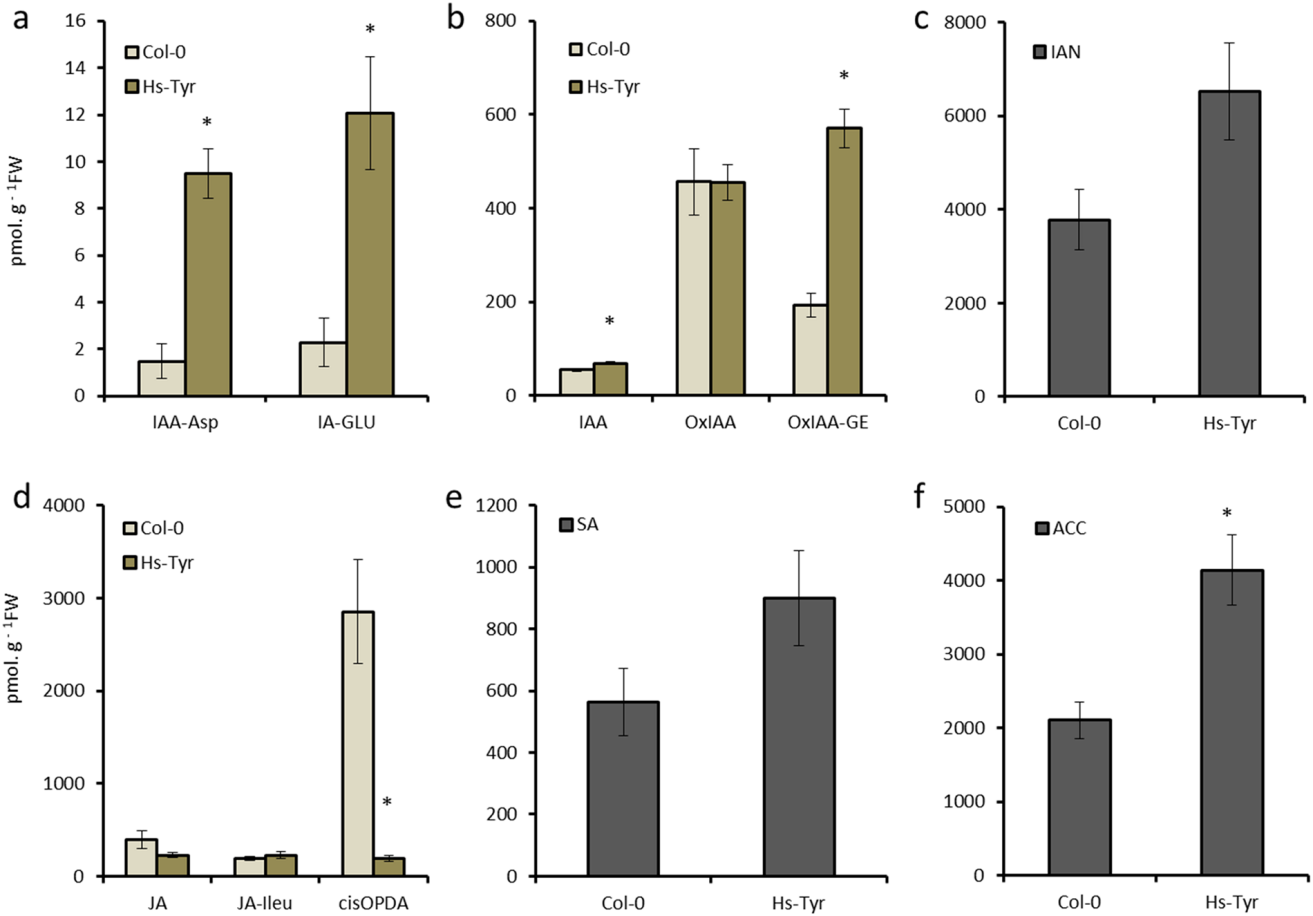
Concentration of various hormones (pmol.g^−1^FW) in roots of transgenic plants ectopically expressing *Hs-Tyr* compared with Col-0. IAA, indole-3-acetic acid; IAA-Asp, IAA-aspartate; IAA-Glu, IAA-glutamate; OxIAA, oxo-IAA; OxIAA-GE = oxo-IAA-glucose ester; IAN, Indole-3-acetonitrile (IAA precursor); of jasmonic acid (JA), JA-isoleucine (JA-Ile), JA precursor *cis*-12-oxo-10,15-phytodienoic acid (cisOPDA), salicylic acid (SA) and 1-aminocyclopropane-1-carboxylic acid (ACC). Each bar represents the mean ± standard error of n = 5. Asterisks indicate significant differences based on Student’s *t-test* (P < 0.05).

To explain the changes of the plant growth, endogenous hormones were measured in *Hs-Tyr* expressing Arabidopsis roots and compared with the wild type Col-0 using HPLC-MS. Roots of line 2.3, which showed the highest susceptibility, were analyzed and compared with Col-0 roots. Results show that the levels of the auxin precursor indole-3-acetonitrile and of IAA metabolites were significantly increased in *Hs-Tyr* expressing plants (Fig. 6a–c). The level of jasmonate precursor *cis*OPDA was lower in the transgenic plants, while no significant change of jasmonic acid and jasmonate isoleucine was observed (Fig. 6d). Concentration of the immediate ethylene precursor 1-aminocyclopropane-1-carboxylic acid (ACC) was higher in the roots of transgenic plants compared with Col-0 (Fig. 6f). No significant changes were detected in the SA concentration (Fig. 6e).

### *Hs-Tyr* localization in *Nicotiana benthamiana* leaves

**Figure 7:**
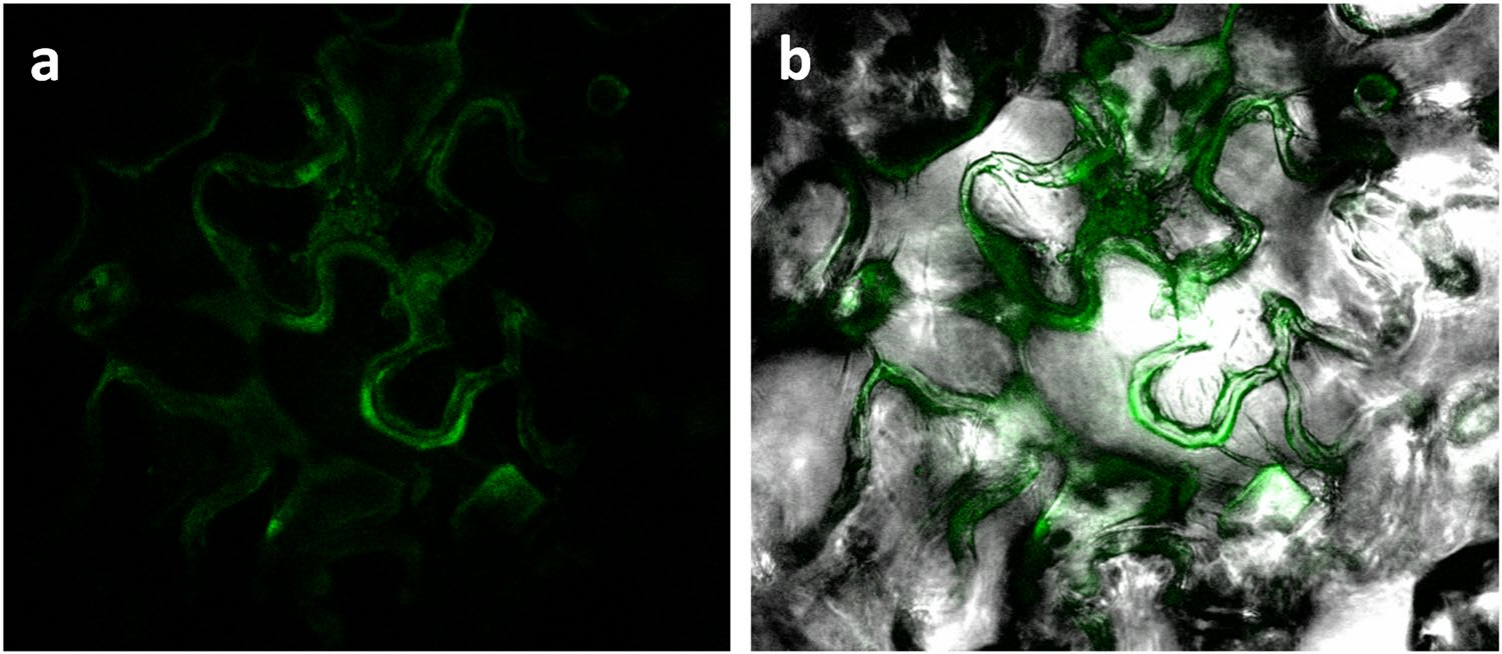
Subcellular localization of *Hs-Tyr*::GFP within *N. benthamiana* leaf epidermal cell. (**a**) The green fluorescence originates from *Hs-Tyr*::GFP fusion protein localized in the cytoplasm of the cells. (**b**) Merged image shows the GFP signal in bright field.

To investigate the action site of *Hs-Tyr* in the plant, a transient transformation of *N. benthamiana* leaves was performed. The leaves were infiltrated with Agrobacterium expressing *Hs-Tyr::GFP* and checked for green fluorescence after 5 days. Results showed that the protein was translated and its fluorescent signal was localized in the cytoplasm of the *N. benthamiana* leaf cells (Fig. 7).

## Discussion

*H. schachtii* can dramatically decrease the yield of sugar beet. Understanding the mechanisms by which nematodes manipulate the plants may give clues in search for novel management strategies. Recent advances in techniques such as RNAseq and next generation sequencing facilitate the mining of plant parasitic nematode genes involved in parasitism^14, 15^. The ability of *H. schachtii* to infect the model host plant *A. thaliana* ^16^ opens additional perspectives in studying gene functions. Using available transcriptomic data of *H. schachtii* we identified a putative effector that may play a role in nematode parasitism. The bioinformatic analysis of the predicted amino acid sequence showed the presence of a signal peptide and a lack of transmembrane domains, which is a strong indication that the protein is secreted. These criteria have been used as standards for screening secreted nematode effectors in other labs^17, 18^. Furthermore, checking for the functional domain(s) showed the presence of a Tyrosinase domain that is, to our knowledge, the first time described in relation to nematode plant-parasitism. Phylogenetic analysis revealed *Hs-Tyr* homologues in plant parasitic, animal parasitic and free-living nematodes. The constructed phylogenetic tree displays the relation of *Hs-Tyr* to other nematodes (Fig. 1). It shows that *Ascaris spp*. is clustering separately from *C. elegans* and plant-parasitic nematode species. Furthermore, cyst nematodes form a subcluster separate from root-knot nematodes within the plant-parasitic nematode cluster. This separation may also hint to a functional divergence and could explain why the *Hs-Tyr* transgenic lines were more susceptible to cyst nematodes but not to root-knot nematodes.

Supported by several experimental approaches we show that *Hs-Tyr* contributes to successful parasitism of the cyst nematode. First, we found that *Hs-Tyr* expression is localized within the dorsal oesophageal gland shown by using whole mount *in situ* hybridization; this suggests that the protein is secreted from the dorsal gland into the plant. Secondly, we showed a dramatic increase in *Hs-Tyr* expression after Arabidopsis infection, which is further linking *Hs-Tyr* expression with a role in parasitism. Knocking down *Hs-Tyr* by RNAi resulted in approximately 80% decrease of *Hs-Tyr* expression. This result is in a range that was found in previous studies on gene silencing by RNAi^19^. The *Hs-Tyr*-silenced nematodes were suffering throughout their life stages as indicated by the small size of females and associated syncytia (Fig. 2). These findings are similar to previous studies, which showed that reduced expression of nematode effectors obstructs nematode development in plants^11, 20^. Furthermore, we show that expressing *Hs-Tyr* in the Arabidopsis plants increased the plant susceptibility to the *H. schachtii*, but not to the root-knot nematode *M. incognita*. We therefore conclude that *Hs-Tyr* functions specifically within a pathway supporting an efficient *H. schachtii* parasitism, while being redundant for *M. incognita*.

We did not observe any hypersensitive responses in the infiltrated *N. benthamiana* leaves and in the transgenic plant, suggesting that *Hs-Tyr* was not recognised by the plant immune system. This assumption is in agreement with the lack of significant changes in JA and SA levels. According to previous analyses, some nematode effectors induced a hypersensitive response, however, *Hs-Tyr* obviously does not belong to this type of effectors^21^.

Interestingly, *Hs-Tyr* expression in the plant caused changes in the plant growth represented by an increasing number of plant lateral roots and shoot weight, both features that may be related to the observed changes in hormone homeostasis. In fact, we showed that auxin biosynthesis was enhanced by *Hs-Tyr* expression in the plant, as indicated by higher content of both auxin precursor and auxin metabolites in the transgenic plant. Auxin homeostasis in roots is well known to determine lateral root formation^22^. Hyper-susceptibility of the *Hs-Tyr* overexpression plants to nematodes could be correlated to the hormonal changes in two ways. First, a high concentration of the plant ACC/ET results in higher attraction of the infective J2s to the roots, which leads to the increase of the number of J2s infecting the plant. This explanation is supported by previous studies, which showed that root exudates of ET-overexpressing mutants are more attractive to nematodes^23^. Furthermore, treating the plant with ethephon as a source of ET, increased attraction of the pre-infective J2s. In contrast, when ACC inhibited by aminooxyacetic acid treatment, the number of females and males developing on the plants was reduced^24^. Secondly, a lower concentration of jasmonic acid increases susceptibility of the transgenic plants towards J2s. In fact, it was shown that JA is a main player during early plant defence against nematode infection. On the other hand, nematodes were shown to trigger up-regulation of plant genes which suppress JA-based defence during the infection^24^. Additionally, the Arabidopsis mutants DELAYED DEHISCENCE 2 (*dde2*) and LIPOXYGENASE 6 (l*ox6*), which are deficient in JA biosynthesis show higher susceptibility towards *H. schachtii* and increased female development compared to wild-type plants^24^. These results indicate that *Hs-Tyr* interferes with plant growth pathways and triggers hormonal changes promoting nematode parasitism^12, 25, 26^.

Taken together, our results clearly identify *Hs-Tyr* as an effector protein produced in the esophageal gland and playing an important role in parasitism of *H. schachtii*. Functional analysis of the protein suggests a mode of action through changes in the homeostasis of plant hormones.

## Methods

### Plant growth and nematode culture

Transgenic lines and the Col-0 wild type *Arabidopsis thaliana* L. Heyn plants were grown aseptically on agar medium supplemented with modified Knop’s nutrient solutions for *H. schachtii* infection and on MS medium for *M. incognita* under conditions described previously^16^.

Mature cysts were collected from white mustard (*Sinapis alba* L.) cvar. Albatros plants in funnels and hatched in 3 mM ZnCl_2_^16^. The freshly hatched pre-parasitic second stage juveniles (J2s) were collected for direct RNA extraction, infecting the Arabidopsis plants for post infective stages collection and for infection assay.

The *M. incognita* J2s were collected from egg masses cultured on tomato grown in the greenhouse. Eggs were isolated from egg masses on tomato roots with 1.5% sodium hypochlorite and rinsed with water on a 25-µm sieve. Eggs were hatched in a solution of 2 ml of gentamycin sulphate (22.5 mg.ml^−1^) and 150 µl of nystatin (10.000 U.ml^−1^) in 30 ml water for 4 days at room temperature in the dark. The hatched nematodes were collected and further surface sterilized as described previously^27^. Briefly, nematodes were surface sterilized by incubation for 20 min in 0.5% (w/v) streptomycin/penicillin solution, for 20 min in 0.1% (w/v) ampicillin/gentamycin solution, for 5 min in sterile tap water, and for 3 min in 0.1% (v/v) chlorhexidine solution. The nematodes were subsequently washed three times in sterile tap water and then used for infection.

### Infection assay

Nematode infection assays on Arabidopsis plants either for the RNA interference (RNAi) experiments or on *Hs-Tyr* overexpression lines were performed for the *H. schachtii* as described before^28^. Briefly, seeds were plated for ten days on 0.2% Knop medium. Plants were inoculated with 60–70 J2 nematodes per plant. Various susceptibility parameters including, number of male and female nematodes per plant was counted after 12 DAI. On the following day, the average sizes of female nematodes and associated syncytia were measured. All experiments were repeated independently three times. Each experiment contained 12 plants per line.

For *M. incognita* infection, ten days old plants on MS media were infected with 100 nematodes per plant. Number and size of galls were collected after 22 days. Ten plants per line and wild type Col-0 were infected. All measures were taken using Leica M165C Binocular (Leica Microsystems, Wetzlar, Germany) and Leica Application Suite software. All experiments were repeated independently three times.

### Sequence analysis and phylogeny

*Hs-Tyr* was determined as one of the predicted putative secreted protein (PSPs) in a *H*. s*chachtii* transcriptome assembly (Elashry *et al*. unpublished). The *Hs-Tyr* sequence was translated using CLC genomics workbench (V8.0) and analysed to predict the longest ORF and functional domain(s) by Pfam database (http://pfam.xfam.org/) and HMMER algorithm^29^, signal peptide by signalP4 server^30^, and transmembrane domain by TMHMM^31^.

*Hs-Tyr* homologues in other nematode species were identified using BLASTP search against the nr database in the NCBI database. Further, we downloaded transcriptomes of *M. incognita* ^32^, *M. hapla* ^33^, *Nacobus aberrans* ^14^, *H. avenae* ^34^ and *G. pallida* ^35^. All transcriptomes were examined by CLC genomics workbench (V8.0) to identify *Hs-Tyr* homologues. All *Hs-Tyr* best hit homologues (see Supplementary Table S1) were analysed structurally to confirm similarities of their functional domains and aligned to each other to build a phylogenetic tree by UPGMA algorithm with distance measured by Jukes-Cantor and 100 bootstraps (CLC genomics workbench V.8.0).

### *In situ* hybridization

Subsequent PCR was performed on gene specific PCR product using primers in Supplementary Table S2 with presence of DIG-labelled deoxynucleotide triphosphates (dNTPs) (Roche). Riboprobes were prepared using single sense primer (negative control) and the antisense primer. The riboprobes hybridized in pre-parasitic J2s as described previously^36^. The hybridized nematodes were visualized using the DMI2000 compound microscope (Leica Microsystems).

### Developmental expression pattern analysis

Real time quantitative PCR was used to analyse the *Hs-Tyr* transcript at different developmental stages of *H. schachtii* using a gene specific primers (Supplementary Table S2). Around 3000 eggs and 3000 pre-parasitic J2s were collected directly from cysts. Around 500–600 nematodes were collected manually by separating them from the *A. thaliana* roots after 5, 10, 15, 20 DAI representing J3s, J4s, females and late females respectively.

RNA was extracted using NucleoSpin RNA kit (MACHEREY-NAGEL) following the manufacture´s protocol. The first strand cDNA was synthesized using the High-Capacity cDNA Reverse Transcription Kit (Applied Biosystems) in presence of the oligo-dT primer. The resulted cDNA were tested for the expression changes using the Stepone Plus Real-Time PCR System (Applied Biosystems) following the amplification conditions: 95 °C for 15 s and 60 °C for 30 s (40 cycles). Each sample contained 10 μl of Fast SYBR Green qPCR Master Mix (Invitrogen), 9 μl of the primer mix with final concentration 1 μM for each primer, 1 μl of cDNA. The amplified data were analysed using one step system to create Ct values. The resulted data were analysed and relative expression was calculated^37^. Actin was used as internal control for all experiments. Three biological replicates from each stage were used with three technical replicates.

### RNA interference and *Hs-Tyr* silencing in nematodes

*Hs-Tyr* specific dsRNA was generated following the manufacturer’s instructions of MEGAscript T7 kit (Ambion, Life Technologies). The GFP DNA fragment was amplified to synthesise dsRNA as a negative control.

Freshly hatched nematodes were soaked for one day in 50 µL soaking mix (1 µg/µL dsRNA (25 µL), 10x soaking buffer (5 µL), 100 mM spermidine (1.5 µL), 500 mM octopamine (5 µL), nematodes in water (13.5 µL)). After that, nematodes were washed three times with sterile water and sterilized using HgCl_2_ for 4 min. Nematodes were washed three times with fresh water. After sterilization, nematodes were divided to two parts. One part was used to evaluate the gene expression after silencing by qPCR. While, the second part was used to infect the Arabidopsis plants as described above.

### Construct generation and *N. benthamiana* agroinfiltration

The *Hs-Tyr* without signal peptide-encoding region was cloned in the binary expression vector pMDC83 using primers in Supplementary Table S2. The pMDC83 vector contains C-terminal GFP fusion protein driven by 2 × 35 S promoter^38^. The *Hs-Tyr::GFP* construct was transformed in *Agrobacterium tumefaciens* strain GV3101::pMP90^39^. The transformed Agrobacterium were grown overnight in 50 ml YEB liquid medium with 10 mg.ml^−1^ gentamycin, 50 mg.ml^−1^ kanamycin and 50 mg.ml^−1^ rifampicin to an OD600 of 0.8 in an incubator/shaker at 28 °C. Bacteria were harvested by centrifugation at 4000 rpm for 7 min at room temperature. The pellet was suspended in infiltration buffer^39^. Bacterial suspensions were diluted in the infiltration buffer to OD600 = 1. After incubation for 2–4 h at RT, Agrobacteria suspensions were infiltrated in the abaxial side of 6 weeks *N. benthamiana* leaves by using 1 ml syringe. For co-infiltration of RNA silencing inhibitor P19, an equal volume of a bacterial suspension harbouring pBin61-P19^40^ was mixed and infiltrated. Infiltrated plants were incubated for 5 days. Slides were made from the infiltrated leaves and tested under the confocal microscope for detecting the green signal in the leaves cells.

### Production of transgenic lines, phenotyping and infection assays

The *Hs-Tyr* ORF without signal peptide was cloned into the binary Gateway overexpression vector pB2GW7^41^. The construct was then transferred to the *A. tumefaciens* strain GV3101, and transformed into *Arabidopsis thaliana* Col-0 using the floral dip method^42^. The seeds of the primary transformants were selected for BASTA resistance (BayerCropScience, Monheim, Germany). In the T2 generation, the lines segregating 3:1 (BASTA-resistant/BASTA-susceptible) were grown to the next generation. Three homozygous lines were selected on BASTA plates and used in the study.

The selected lines were grown on MS plates for 10 days, then several phenotypes were measured and compared with the wild type plants Col-0 including the number of the lateral roots, the main root length, the fresh root weight and the fresh shoot weight. The experiment was repeated 3 times and each experiment consists of 9 plants for each line. The selected lines were subjected as well to the infection of the nematodes *H. schachtii* and *M. incognita* as mentioned in the infection assay section.

### Hormone analysis

Root samples were collected from ten-days old plants. Five root samples (150 mg each) were collected from *Hs-Tyr*-expressing plants (Line 2.3) or Col-0. Root samples were purified and analysed as mentioned previously^43, 44^. Briefly, samples were homogenized with a ball mill (MM301, Retsch) and extracted in cold (−20 °C) methanol/water/formic acid (15/4/1 v/v/v). The following labelled internal standards (10 pmol/sample) were added: ^13^C_6_-IAA (Cambridge Isotope Laboratories); ^2^H_4_-SA (Sigma-Aldrich); ^2^H_2_-OxIAA and ^2^H_5_-JA(Olchemim). Extracts were purified using SPE-C18 column (SepPak-C18, Waters) and a mixed mode reverse phase–cation exchange SPE column (Oasis-MCX, Waters). Hormone metabolites were analysed using HPLC (Ultimate 3000, Dionex) coupled to a hybrid triple quadrupole/linear ion trap mass spectrometer (3200 Q TRAP, Applied Biosystems). Quantification of hormones was done using the isotope dilution method with multilevel calibration curves (r^2^ > 0.99). Data processing was carried out with Analyst 1.5 software (Applied Biosystems). Data are presented as mean ± standard error.

### Statistical analysis

To test significant differences between the variants, Student’s *t*-test was used and data represented as mean values ± standard error. *P* < 0.05 was used to determine significance.

## Electronic supplementary material

Supplementary Info
