# Supplementary material for: Heterodera schachtii Tyrosinase-like protein - a novel nematode effector modulating plant hormone homeostasis
Source: Sci Rep. 2017 Jul 31;7:6874. doi: 10.1038/s41598-017-07269-7 (PMC5537230; doi:10.1038/s41598-017-07269-7)
Supplement: Supplementary file 1 — Supplementary Info [file 41598_2017_7269_MOESM1_ESM.doc]

***Heterodera schachtii* Tyrosinase-like protein - a novel nematode effector modulating plant hormone homeostasis**

Samer S. Habash^1^, Zoran S. Radakovic^1^, Radomira Vankova^2^, Shahid Siddique^1^, Petre Dobrev^2^, Cynthia Gleason^3^, Florian M.W. Grundler^1*^, Abdelnaser Elashry^1^

^1^Rheinische Friedrich-Wilhelms-University of Bonn, INRES – Molecular Phytomedicine, Karlrobert-Kreiten-Straße 13, D-53115 Bonn, Germany

^2^Institute of Experimental Botany AS CR, Rozvojová 263, CZ-16502 Prague 6, Czech Republic.

^3^ Washington State University, Dept. of Plant Pathology, Pullman WA 99164-6430, 335 Johnson Hall, USA

Author for correspondence:

*Florian M.W. Grundler

Tel: 00490228731675

E-mail: [grundler@uni-bonn.de](mailto:grundler@uni-bonn.de)

**Supplementary information**

**
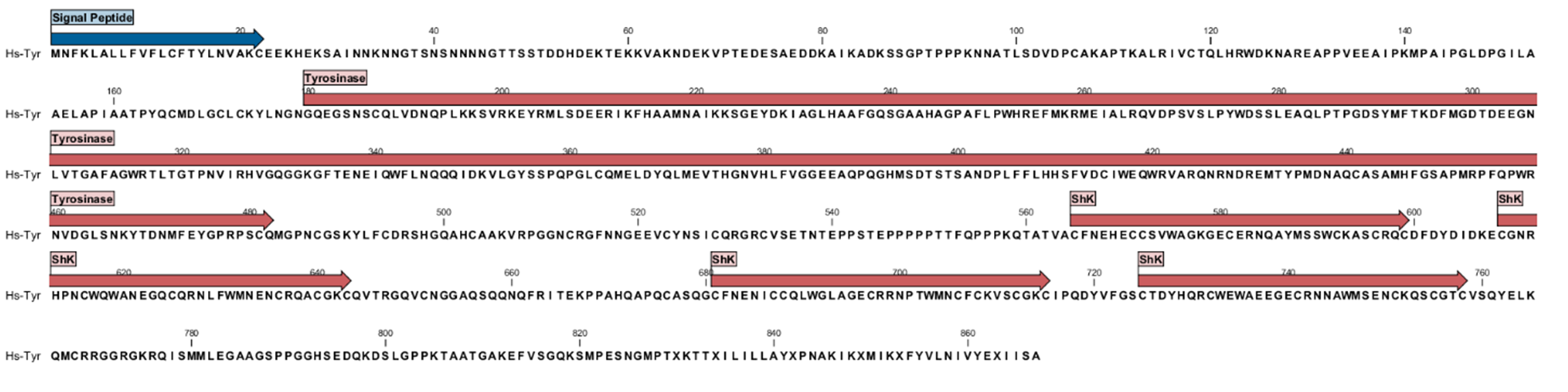
**

**Supplementary Fig. S1. Structure and functional annotation of *Hs-Tyr*.** Detailed overview of amino acid sequence of *Hs-Tyr* with predicted signal peptide (Blue), Tyrosinase doman and ShK domains domains (pink). Prediction was done using signalP4 server, Pfam database (<http://pfam.xfam.org/>) and CLC genomics workbench (V8.0).


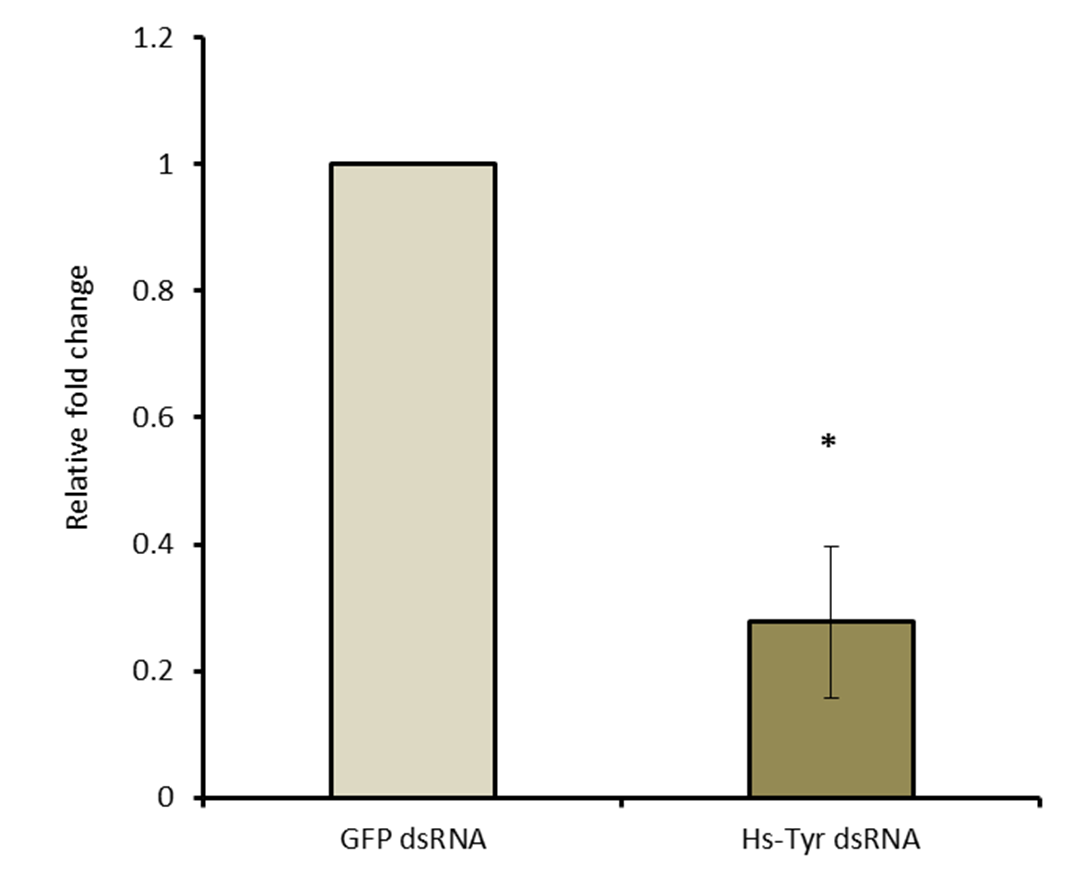


**Supplementary Fig. S2. Silencing of *Hs-Tyr* expression in J2s via RNA interference.** *Hs-Tyr* expression was measured after incubating the J2s in *Hs-Tyr* dsRNA and compared with the J2s were incubated in GFP dsRNA as control. Data are based on three independent experiments. Each bar represents the mean ± standard error of n =9.


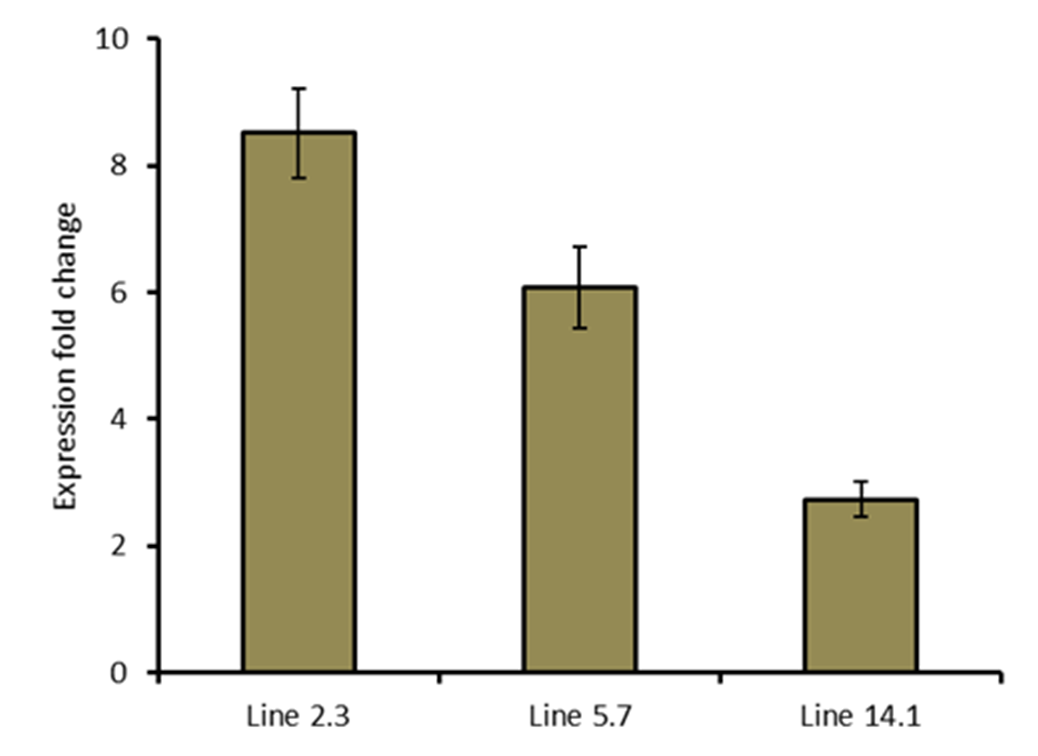


**Supplementary Fig. S3. Relative mRNA expression level of *Hs-Tyr* in the transgenic Arabidopsis lines.** Data are based on three independent experiments. Each bar represents the mean ± standard error of n =9. The plant actin was used as an internal control to normalize gene expression level.


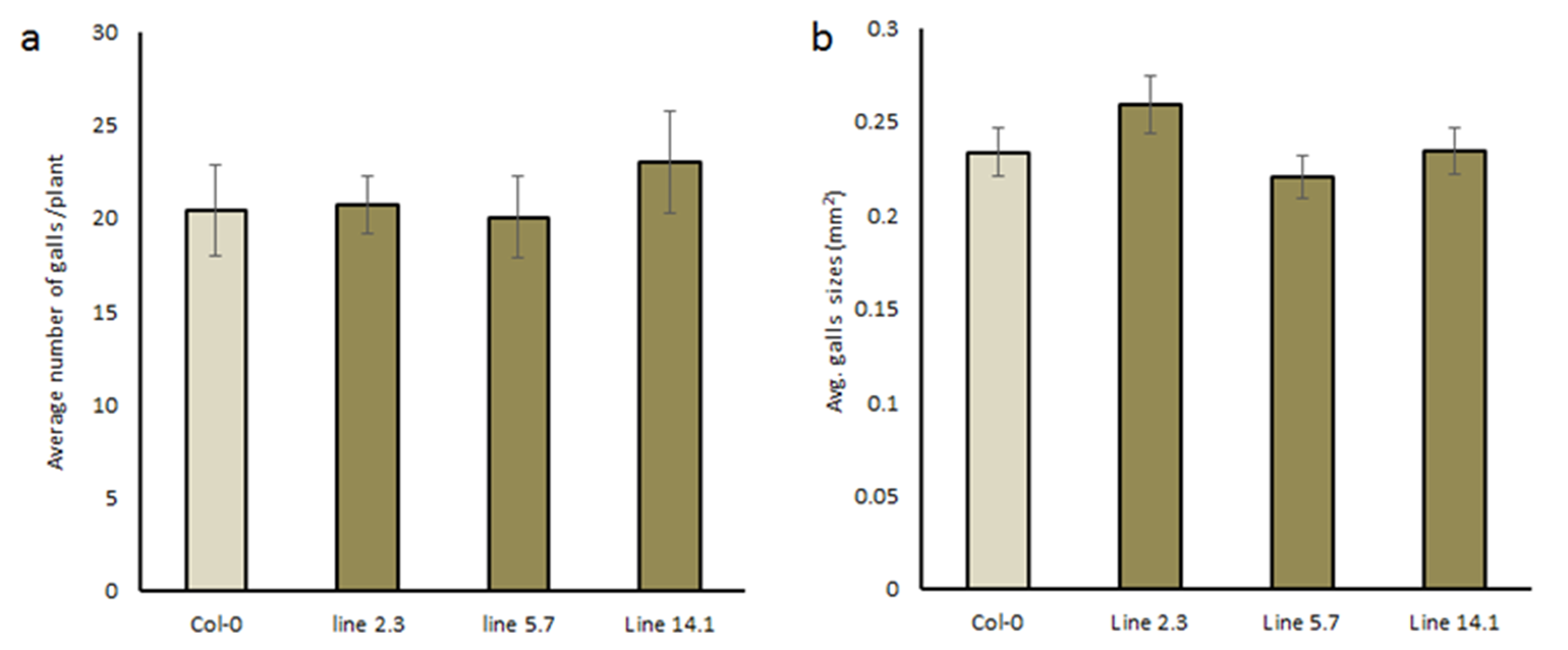


**Supplementary Fig. S4. Overexpressing the *Hs-Tyr* did not affect the *M. incognita* infection on Arabidopsis.** Transgenic plants didn’t show any susceptibility towards nematode parasitism compared with the wild type Col-0 plants represented by (a) Average number of galls per plant (b) Average size of nematode galls. Data are based on three independent experiments. Each bar represents the mean ± standard error of n = 30. Asterisk marks indicates significant differences based on Student's *t-test* (P < 0.05).

**
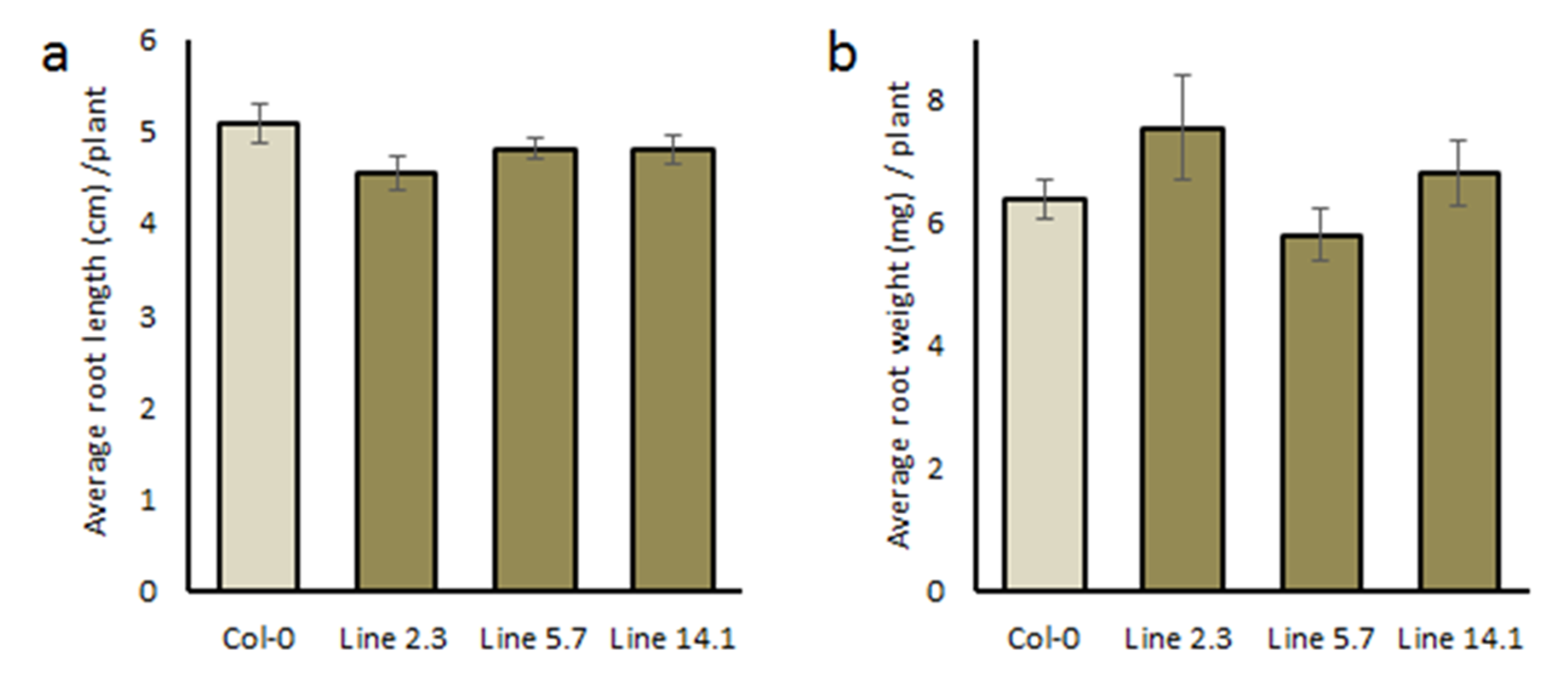
**

**Supplementary Fig. S5. Phenotype of the transgenic Arabidopsis plants root ectopically expressing *Hs-Tyr*.** (a) root length per plant (b) root weight per plant, compared with the wild type Col-0. Data represent average of three independent experiments with nine plants per each. Data are based on three independent experiments. Each bar represents the mean ± standard error of n =27. Asterisk marks indicates significant differences based on Student's *t-test* (P < 0.05).

**Supplementary Table S1 Sequence names and accession numbers that were used in the phylogeny analysis**

| **Species** | **Contig name/ Accession number** | **Source** |
| --- | --- | --- |
| *Meloidogyne incognita* | Minc10168b | Abad *et al.* 2008 |
| *Meloidogyne incognita* | Minc10168a | Abad *et al.* 2008 |
| *Meloidogyne incognita* | Minc08903 | Abad *et al.* 2008 |
| *Meloidogyne hapla* | Contig225.frtz3.gene3 | Opperman *et al.* 2008 |
| *Nacobbus aberrans* | Nab_25123_c0_seq1 | Eves-van den Akker et al.2014 |
| *Heterodera avenae* | Locus_2044 | Kumar *et al.* 2014 |
| *Globodera pallida* | GPLIN_000659000 | Cotton *et al.* 2014 |
| *Heterodera schachtii* | *Hs-Tyr* (C12694) | Elashry *et al.* Unpublished |
| *Ascaris lumbricoides* | ALUE_0001861501 | NCBI |
| *Ascaris suum* | F1KXGS_ASCSU | NCBI |
| *Caenorhabditis elegans* | NP_491709 | NCBI |
| *Caenorhabditis elegans* | NP_492055 | NCBI |

**Supplementary T**able S2 Primer names and sequences used in the study

| **Primer Lable** | **Primer sequence** |
| --- | --- |
| Tyr-In situ-F | TCCGCCGACAACATTCCA |
| Tyr-In situ-R | TGATGCGCTGGTGGTTTT |
| Tyr-qPCR- F | ACAAGCATGCGGAAAGTG |
| Tyr-qPCR- R | TGATGCGCTGGTGGTTTT |
| Hs-Actin-F | CGTGACCTCACTGACTACCT |
| Hs-Actin-R | CGTAGCACAACTTCTCCTTG |
| RNAi-F | TAATACGACTCACTATAGGGAGA AGCGACGAAGAACGAATC |
| RNAi-R | TAATACGACTCACTATAGGGAGA GTGTCGCCCATGAAATCT |
| Loc Fw | GAAGAAAAGCATGAAAAATC |
| Loc Rw | TGTTGGCATTCCGTTACT |
| At-Actin-F | ACAGCAGAGCGGGAAATTGT |
| At-Actin-R | AGCAGCTTCCATTCCCACAA |
